# Sleep quality was associated with adverse reactions after coronavirus disease 2019 vaccination among healthcare workers: A longitudinal paired study

**DOI:** 10.3389/fnbeh.2022.867650

**Published:** 2023-01-04

**Authors:** Ning Xiao, Xingli Xu, Zhiyue Ma, Xiaoxu Yu, Yong Feng, Bilan Li, Yuping Liu, Gang He, Jiangang Fan, Bin Li, Xiaolong Zhao

**Affiliations:** ^1^Department of Otolaryngology Head and Neck Surgery, Sichuan Provincial People’s Hospital, Chinese Academy of Sciences Sichuan Translational Medicine Research Hospital, University of Electronic Science and Technology of China, Chengdu, China; ^2^Department of Health Management Center and Institute of Health Management, Sichuan Provincial People’s Hospital, Chengdu, China; ^3^Ultrasound in Cardiac Electrophysiology and Biomechanics Key Laboratory of Sichuan Province, Sichuan Provincial People’s Hospital, Chinese Academy of Sciences Sichuan Translational Medicine Research Hospital, University of Electronic Science and Technology of China, Chengdu, China

**Keywords:** COVID-19, vaccine, sleep disorder, adverse reactions, healthcare workers

## Abstract

**Background:**

Many countries have currently relied on various types of vaccines for the public to control the coronavirus disease 2019 (COVID-19) pandemic. The adverse reactions (ARs) after vaccination may affect vaccination coverage and confidence. However, whether sleep quality was associated with ARs after vaccination remains unclear.

**Methods:**

We designed a longitudinal paired study within a hospital setting. We collected data about the side effects within 7 days after two doses of scheduled vaccination among healthcare workers (HCWs). All HCWs were asked to complete a sleep survey indexed by the Pittsburgh Sleep Quality Index (PSQI) before vaccination and after a 1-month follow-up. Then, we explored the relationship between sleep quality before or after vaccination and the occurrence of ARs.

**Results:**

A total of 345 HCWs were recruited to receive COVID-19 vaccination. The sleep quality became worse after vaccination. All local and systemic reactions were mild or moderate in severity (32.46%), and no serious adverse event was reported. Binary logistic regression showed participants with poor sleep quality (PSQI > 5) than good sleep quality (PSQI ≤ 5) before the two doses of vaccination, respectively, exhibited 1.515 and 1.107 times risk of ARs after each vaccination (both *p* < 0.001).

**Conclusion:**

There is an apparently complex bidirectional relationship between sleep quality and COVID-19 vaccination adverse effects. Poor sleep quality significantly increases the risk of mild ARs after vaccination, while vaccination may cause a temporary decline in sleep quality.

## Introduction

The ongoing coronavirus disease 2019 (COVID-19) pandemic caused by severe acute respiratory syndrome coronavirus 2 (SARS-CoV-2) has led to high morbidity and mortality worldwide (Mehra et al., 2020). To control the COVID-19 pandemic, various kinds of vaccines have been developed within an extraordinarily swift timeframe (Garcia and Cerda, 2020). Chinese Food and Drug Administration (FDA) gradually has authorized three types of inactivated vaccines for many countries and regions to prevent SARS-CoV-2 infection, which showed to have approximately 50.65–91.25% efficacy in preventing COVID-19 illness in healthy people, whereas no serious adverse event was reported post-vaccination (Xia et al., 2020, 2021; Zhang et al., 2021). The first phase of the vaccination program in China was primarily focused on healthcare workers (HCWs) nationwide and on long-term care facility residents (who are at the forefront with more viral exposure but took the vaccine as a challenge). Jobs involve shifting work time. This, inevitably, can be stressful and may lead to acute sleep deprivation. The prevalence of sleep disturbances among Chinese healthcare professionals is reported to be approximately 39%, which was higher than the general population (Qiu et al., 2020).

Sleep dysfunction could impose some negative consequences as a result of disturbed mood, daytime dysfunction, and medical errors, absenteeism in HCWs (Parry et al., 2018). Our previous study performed on HCWs to screen for patients with 2019-nCoV has proven that HCWs experienced a poor subjective quality of sleep (Zhao et al., 2020). The public receiving influenza vaccine might experience some sleep dysfunction after influenza vaccination (Sarkanen T. O. et al., 2018). On the contrary, as suggested by many previous studies, the change in sleep quality before and after vaccination did have the potential to impact the immune responses to infectious diseases such as hepatitis A virus and influenza virus (Spiegel et al., 2002; Lange et al., 2011; Prather et al., 2012). It seems that variations in sleep quality also had some impact on the immune responses to vaccination against COVID-19 (Kow and Hasan, 2021). In the natural process of developing immunity, sleep quality before or after vaccination was thus likely an important factor to be associated with the occurrence of adverse reactions (ARs) (Chitkara et al., 2013). The safety and immunological responses for the COVID-19 vaccine were reported and measured by the occurrence of local and systemic ARs (Zhang et al., 2021). In addition, these ARs would likely affect vaccine attitudes and even could destroy public confidence in the immunization program, since these people could experience ARs or worry about additional economic burdens due to ARs (Karafillakis et al., 2016; Smith et al., 2017; Luyten et al., 2019).

Given the urgency of achieving effective global COVID-19 vaccination coverage (Dror et al., 2020; Wadman, 2020), we strongly advocate gathering information about individuals’ sleep patterns before and after vaccination. Thus, we could explore some interaction effects between sleep quality and side effects after vaccination may increase our understanding of the pathophysiology and yield more effective strategies to reduce the risk of side effects after vaccination.

## Materials and methods

### Study population and study procedures

In China, the COVID-19 vaccines are administered by the National Institute of Public Health, part of the Ministry of Health, and the vaccination was conducted in the designated vaccine sector. HCWs in our hospital participants were designated to complete the COVID-19 vaccines in our hospital. Eligible participants were people aged 18–60 years, who were negative for serum-specific IgM/IgG antibodies against SARS-CoV-2, as measured using a commercial kit (Innovita, Tangshan, China). Exclusion criteria were a history of traveling to regions with reported COVID-19 cases from December 2019, regions outside of China, or a history of infection with SARS-CoV; fever, cough, runny nose, sore throat, diarrhea, dyspnea, or tachypnea in the 14 days before vaccination; abnormalities in laboratory tests for SARS-CoV-2 specific IgG or IgM positive in serum; positive PCR test for SARS-CoV-2 from a pharyngeal or anal swab sample; and allergy to any ingredient included in the vaccine.

During the whole of January 2021, a total of 380 eligible participants voluntarily consented to receive COVID-19 vaccines. We personally contacted these HCWs, invited them to participate, and clearly explained the aims and significance of this study, as well as the method by which to complete the study (i.e., sleep survey); however, 20 HCWs refused to participate. The inactivated vaccine was developed by the Beijing Institute of Biological Products (Beijing, China) and manufactured as previously described (Xia et al., 2021). On days 0 and 30, vaccine recipients received inactivated vaccines containing 4 μg of total proteins based on the vaccination schedule. The time for the first dose of vaccination was set as day 0. The first sleep quality was measured with the Pittsburgh Sleep Quality Index (PSQI) questionnaire on day 0. The second sleep quality assessment was taken 1 month later since the first dose of vaccination (day 30). Finally, we checked all data to avoid errors and ensure quality, and there were 15 invalid HCWs (4.16%). Ultimately, we used the data of 345 participants in the present study.

To explore the relationship between the change in sleep quality and whether ARs occurred after both vaccinations, we divided all participants into four subgroups according to whether ARs had occurred or not after both vaccinations and compared their change in sleep quality as follows: a subgroup with no ARs after both vaccinations (*n* = 233); the subgroup with ARs after the first vaccination but none after the second one (*n* = 29); the subgroup with no ARs after the first vaccination but ARs after the second one (*n* = 41); and the subgroup with ARs after both vaccinations (*n* = 42). The participants reported any ARs within the first 7 days after vaccination. Later, we monitored the occurrence of local and systemic ARs and managed them if necessary. All ARs’ categories and treatment methods were followed using the guideline issued by the China State Food and Drug Administration (version 2019) (Wu et al., 2015).

**FIGURE 1:**
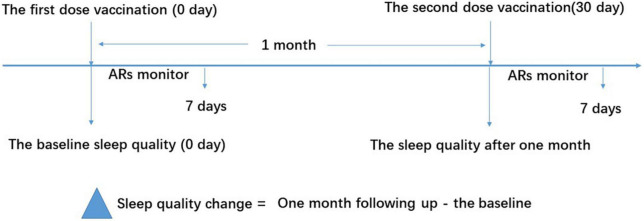
Study flow diagram. The vaccine recipients received two doses of vaccination based on the schedule on days 0 and 30. The sleep quality was measured using the Pittsburgh Sleep Quality Index (PSQI) questionnaire on days 0 and 30 for the baseline and 1-month follow-up, respectively. ARs were monitored within the first 7 days after vaccination. ARs, adverse reactions.

The study flowchart is shown in Figure 1. This study was approved by the Internal Review Board of the Institutional Ethics Committee of Sichuan Provincial People’s Hospital and was conducted in accordance with the Declaration of Helsinki. Written informed consent was obtained from each participant before enrollment.

### Adverse reaction monitor

To guarantee that vaccine-related ARs could be estimated with maximal precision, all participants were investigated for at least 1 h after each injection in the designed vaccine sector in our hospital. After they left the sector, adverse events were self-reported by participants, which were verified by medical investigators. Medical investigators would evaluate these adverse events, including their pathogenesis and possible causal relationships with the vaccine. Once some serious ARs happened, the participant could inform medical investigators or reach the nearest health center or other health facilities as soon as possible.

### Sleep quality assessment

The Pittsburgh Sleep Quality Index assessment is a 19-item self-report instrument, designed to measure sleep quality and disturbances over a 1-month time interval (Jaradat et al., 2020). The 19 items of the questionnaire generate seven “component” scores, i.e., subjective sleep quality, sleep latency, sleep duration, habitual sleep efficiency, sleep disturbances, use of sleeping medication, and daytime dysfunction. The component scores range from 0 (indicating no difficulty) to 3 (indicating severe difficulty). The sum of the seven components yields one PSQI score. These scores are added for a total score between 0 and 21 with higher scores representing lower sleep quality. A PSQI score of > 5 distinguishes patients having poor sleep quality, while a PSQI score of ≤ 5 indicates good sleep.

### Statistics

Data are presented as means (standard deviation, SD), medians (interquartile range), or numbers (percentages), according to whether they had a normal distribution, skewed distribution, or were categorical, respectively. Differences in baseline characteristics among subgroups were examined using the Kruskal–Wallis *H*-test, one-way analysis of variance, Fisher’s exact test, or the χ^2^ test according to the data distribution. The sleep quality change of the participants between the first and second doses of vaccination was examined using the paired Student’s *t*-test, the Wilcoxon sign-rank test, the Kruskal–Wallis test, or the χ^2^ test, as appropriate. We adopted two-way repeated measures ANOVA design to evaluate the difference between subgroups on sleep quality change for baseline and follow-up. The sleep quality for baseline and follow-up was repeatedly measured in our study as the independent variable. In addition, time (baseline and follow-up) and whether there are ARs after vaccination (yes and no) (2 × 2) are the dependent variables used in the repeated-measures ANOVA model. The F statistic is used to test the significance of each effect (i.e., time effect, group effect, and interaction effect). Then, binary forward logistic regression analyses were performed to determine which risk factors were independently associated with the occurrence of ARs after each dose of COVID-19 vaccination, which was adjusted for some demographic factors. All analyses were performed using SPSS software (version 20.0; SPSS Inc., Chicago, IL, USA). *p*-values of < 0.05 were considered significant.

## Results

### Baseline characteristics of healthcare workers in this study

A total of 306 eligible HCW participants who voluntarily agreed and consented to receive COVID-19 vaccines were automatically included in this study. Among these, we excluded 15 workers for some reason (refer to the Section “Materials and methods”), and we obtained data from 345 participants to complete a sleep quality survey and collect AR data (the response rate was 90.78%). Most participants were under the age of 50 years (78%) and were men (74.5%). The mean shift days per month for all participants were 3.5 days. The work time for about half of the participants was above 10 years. With regard to the educational level, marital status, professional title level, and alcohol and smoking habits, the characteristics of the participants are shown in Table 1.

**TABLE 1:** Demographic characteristics of the healthcare workers to receive the COVID-19 vaccine.

	*n* = 345
Demographics	
Age, years	37.29 ± 0.66
No. of males, *n* (%)	257 (74.5)
No. of doctors, n (%)	278 (74.5)
BMI, kg/m^2^	22.32 ± 0.19
Marital status, *n* (% married)	241 (80.57)
Educational level (%)	
Below university	115 (33.3)
University	181 (52.5)
Postgraduate	49 (14.2)
Nightshift days per month	3.50 ± 0.24
Professional title level, *n* (%)	
Junior	230 (66.7)
Mediate	61 (17.7)
Senior	54 (15.7)
Years of work, years	13.40 ± 0.65
Current smoker, *n* (%)	34 (10)
Alcohol drinker, *n* (%)	36 (11)

Continuous data are presented as the means ± standard deviation (SD) and categorical data are presented as numbers with percentages in parentheses.

### Sleep quality before and after vaccination

The Pittsburgh Sleep Quality Index assessment is designed to measure sleep quality over a 1-month time interval. Sleep quality results before the first dose of vaccination were measured on the first dose of vaccination day (day 0) as baseline sleep quality. During the 1-month follow-up period after the first dose of vaccination, 7 PSQI components and total score were repeatedly measured, and their change between baseline and after the 1-month follow-up was calculated (Table 2). Compared with baseline sleep quality, the paired Student’s *t*-test showed significantly higher scores in the follow-up on sleep quality (0.32 ± 0.031 vs. 0.45 ± 0.033, *p* = 0.001), sleep latency (0.66 ± 0.052 vs. 0.95 ± 0.046, *p* < 0.001), sleep duration (0.66 ± 0.041 vs. 1.01 ± 0.042, *p* < 0.001), sleep efficiency (0.70 ± 0.053 vs. 1.41 ± 0.056, *p* < 0.001), sleep disturbances (1.62 ± 0.028 vs. 1.80 ± 0.030, *p* < 0.001), and PSQI score (4.73 ± 0.132 vs. 6.41 ± 0.158, *p* < 0.001). However, the components of the use of medication and daytime dysfunction did not show a significant change (*p* = 0.860 and *p* = 0.890) (Table 2). These results demonstrated that the HCWs had experienced more high percentage of poor sleep after vaccination during the follow-up month (PSQI > 5) (42.0 vs. 60.0%).

**TABLE 2:** Comparison of sleep quality scores of healthcare workers measured using the Pittsburgh Sleep Quality Index (PSQI) assessment before and after vaccination.

	Baseline sleep quality	1 month following up	The change in sleep quality	*P*-value
Sleep quality	0.32 ± 0.03	0.45 ± 0.03	0.133 ± 0.04	0.001
Sleep latency	0.66 ± 0.05	0.95 ± 0.05	0.290 ± 0.07	<0.001
Sleep duration	0.66 ± 0.04	1.01 ± 0.04	0.350 ± 0.06	<0.001
Sleep efficiency	0.70 ± 0.05	1.41 ± 0.06	0.716 ± 0.06	<0.001
Sleep disturbances	1.62 ± 0.03	1.80 ± 0.03	0.177 ± 0.04	<0.001
PSQI	4.73 ± 0.13	6.41 ± 0.16	1.110 ± 0.13	<0.001
Use of medication	0.21 ± 0.03	0.21 ± 0.04	0.009 ± 0.05	0.860
PSQI	4.73 ± 0.13	6.41 ± 0.16	1.110 ± 0.13	<0.001
Daytime dysfunction	0.56 ± 0.04	0.57 ± 0.05	0.009 ± 0.06	0.890
PSQI	4.73 ± 0.13	6.41 ± 0.16	1.110 ± 0.13	<0.001

The data are presented as the means ± SD. Scores obtained before and after 1 month following up were compared using the paired Student’s *t*-test.

### Adverse reactions after each dose of vaccination

Adverse reactions after the first and second doses of vaccination are shown in Table 3. A total of 71 (20.57%) participants reported at least one AR within the first 7 days after the first dose of vaccination: the most common injection site AR was pain, which was reported in 46 (13.4%) participants. The overall most common systematic ARs were muscle pain or arthritis or joint pain (22 [6.4%]) and fatigue or muscular weakness (20 [5.8%]); the participants reported that any ARs within the first 7 days after the second dose of vaccination was 84 (24.34%), and the most common injection site AR was pain, which was reported in 41 (11.9%) participants. The most common systematic AR after the second dose of vaccination was reported as fever in 33 participants (9.6%). Most ARs were mild or moderate in severity. These reactions occurred within 24 h postvaccination and persisted for not more than 48 h. No more new adverse event was self-reported within 28 days.

**TABLE 3:** Adverse reactions within 7 days after vaccination.

	After the first dose vaccination	After the second dose vaccination	Overall adverse reactions
The time when the adverse reactions occurred (days)	1.32 ± 0.514	1.05 ± 0.223	
**All adverse reactions within 7 days**			
Any	71 (20.57%)	84 (24.34%)	112 (32.46%)
Grade 3	2 (0.57%)	2 (0.57%)	4 (1.2%)
**Injection site adverse reactions within 7 days**			
Pain	46 (13.4%)	41 (11.9%)	83 (24%)
Tenderness	40 (11.6%)	17 (4.9%)	51 (14.7%)
Induration or swelling	15 (4.3%)	10 (2.9%)	22 (6.4%)
Rash or redness	13 (3.8%)	20 (5.8%)	30 (8.7%)
Itch	9 (2.6%)	7 (2%)	16 (1.7%)
Cellulitis	3 (0.9%)	3 (0.9%)	6 (1.7%)
**Systemic adverse reactions within 7 days**			
Fever	3 (0.9%)	33 (9.6%)	36 (10.4%)
Tachycardia	3 (0.9%)	8 (2.3%)	8 (2.3%)
Bradycardia	13 (3.8%)	12 (3.5%)	14 (4.1%)
Hypertension	9 (2.6%)	7 (2%)	9 (2.6%)
Drop of blood pressure	9 (2.6%)	7 (2%)	9 (2.6%)
Tachypnea	20 (5.8%)	18 (5.2%)	26 (7.6%)
Diarrhea	6 (1.7%)	6 (1.7%)	6 (1.7%)
Constipation	6 (1.7%)	3 (0.9%)	6 (1.7%)
Dysphagia	6 (1.7%)	3 (0.9%)	6 (1.7%)
Appetite impaired	6 (1.7%)	4 (1.2%)	6 (1.7%)
Vomiting	3 (0.9%)	19 (5.5%)	19 (5.5%)
Nausea	3 (0.9%)	16 (4.6%)	19 (5.5%)
Muscle pain or arthritis or joint pain	22 (6.4%)	25 (7.2%)	32 (9.3%)
Headache or dizziness or new convulsions	16 (4.7%)	18 (5.2%)	22 (6.4%)
Cough	11 (3.2%)	17 (4.9%)	20 (5.8%)
Dyspnea	4 (1.2%)	3 (0.9%)	7 (2%)
Acute anaphylaxis or mucosal abnormality	0	3 (0.9%)	3 (0.9%)
Fatigue or muscular weakness	20 (5.8%)	3 (0.9%)	23 (6.7%)

Continuous data are presented as the means ± standard deviation (SD) and categorical data are presented as numbers with percentages in parentheses. Any refers to all the participants with any grade adverse reactions or events. Adverse reactions and events were graded according to the scale issued by the China State Food and Drug Administration. Grade 3 = severe (i.e., prevented activity).

### Relationship between sleep quality before vaccination and the chance of adverse reactions after vaccination

To explore the relationship between the sleep quality before each dose of vaccination and ARs after each dose of vaccination, the binary forward logistic regression analysis was performed and shown in Table 4. The higher baseline PSQI score, indicative of worse sleep quality, was associated with a higher chance of ARs after the first dose of vaccination (OR, 1.515; 95% CI: 1.326, 1.731, *p* = 0.001). The 1-month follow-up PSQI score rather than the baseline PSQI was positively correlated with the ARs after the second dose of vaccination (OR, 1.107; 95% CI: 1.108, 1.203, *p* = 0.016). In addition, the baseline PSQI before the first dose of vaccination was associated with the overall ARs after both vaccinations (OR, 1.147; 95% CI: 1.043, 1.261, *p* = 0.005). All regression models were adjusted for demographic factors including age, BMI, nightshift days per month, and years of work, as well as sex, marital status, type of work, smoking status, drinking status, educational level, and professional title.

**TABLE 4:** The relationship between the sleep quality before vaccination and after vaccination and adverse reactions (ARs) after vaccination in healthcare workers.

	ARs after first dose vaccination		ARs after second dose vaccination		Overall ARs	
	OR (95% CI)	*P*-value	OR (95% CI)	*P*-value	OR (95% CI)	*P*-value
The baseline PSQI	1.515 (1.326,1.731)	0.001	NS	NS	1.147 (1.043,1.261)	0.005
The 1-month following up PSQI	NS	NS	1.107 (1.018,1.203)	0.016	NS	NS

The data are presented as the odds ratio (OR) (95% CI). NS, not significant. We performed binary forward logistic regression to determine the potential sleep factors associated with the adverse reactions after vaccination during follow-up. The regression model was adjusted for age, BMI, nightshift days per month, and years of work (continuous variables), as well as sex, marital status, type of work (doctor or not), smoking status, drinking status, educational level, and professional title (categorized variables).

### The role of the sleep quality change between before and after vaccination in the chance of adverse reactions

**FIGURE 2:**
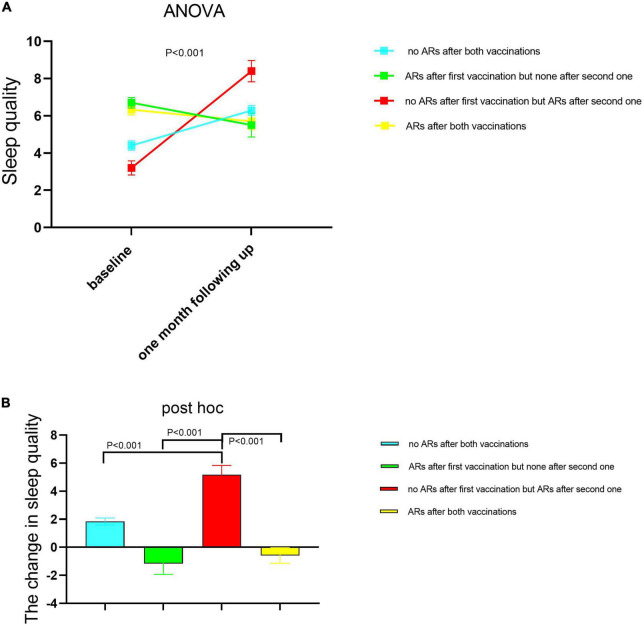
The sleep quality changed between baseline and 1-month follow-up among the four healthcare worker (HCW) subgroups according to whether having ARs or not after each dose of vaccination. A subgroup with no ARs after both vaccinations (*n* = 233); the subgroup with ARs after the first vaccination but none after the second one (*n* = 29); the subgroup with no ARs after the first vaccination but ARs after the second one (*n* = 41); the subgroup with ARs after both vaccinations (*n* = 42). The sleep quality was measured before each dose of injection. The second sleep quality measurement after the first dose of injection was the corresponding one before the second dose of injection. The difference was compared in sleep quality among these four subgroups using one-way ANOVA **(A)**, and the *post hoc* was performed between each two subgroups respectively **(B)**. Then, AR monitoring data after each vaccine injection was collected. The data are presented as the means ± SD. Differences in sleep quality among four subgroups were examined using the one-way analysis of variance. PSQI scores with higher scores represented lower sleep quality. PSQI, Pittsburgh Sleep Quality Index; ARs, adverse reactions.

We divided all participants into four subgroups based on whether ARs occurred or not after both vaccinations as specified in the Section “Materials and methods.” We compared the difference in sleep quality among these four subgroups using one-way ANOVA (Figure 2A) and found that sleep quality was significantly different among the four subgroups in baseline and 1-month follow-up, respectively. Demographic characteristics among four subgroups were shown in Table 5. Then, the repeated measures ANOVAs were conducted. We found that there was a significant main effect for time on sleep quality (baseline vs. following up) [(*F* = 20.85,1) = 0.058, *p* < 0.001]. There were some significant interactions between subgroups and time [(*F* = 21.11,3) = 0.157, *p* < 0.001], which means that the sleep quality uniformly varied depending on the different subgroups. For the baseline sleep quality, *post-hoc* pairwise comparison revealed that PSQI was significantly less for the subgroup with no ARs after the first vaccination but ARs after the second vaccination (red subgroup) (3.26 ± 0.38) compared with the subgroup with no ARs after both vaccinations, the subgroup with ARs after first vaccination but none after the second vaccination, and the subgroup with ARs after both vaccinations (4.4 ± 0.25; 6.7 ± 0.28; 6.33 ± 0.28; *p* = 0.000). By following up on sleep quality, *post-hoc* analysis revealed that PSQI was significantly highest for the same subgroup (red subgroup), including with no ARs after the first vaccination but ARs after the second one, then the other three subgroups (8.4 ± 0.57 vs. 6.2 ± 0.26; 5.5 ± 0.64; 5.7 ± 0.44; *p* = 0.000). To clearly delineate the effect of sleep quality on the occurrence of ARs, we calculated the difference in sleep quality before and after vaccination among four subgroups. We found that the participants who experienced worse sleep quality change after the first dose of vaccination may have a higher chance of ARs after the second dose of vaccination even though without ARs after the first dose of vaccination (Figure 2B). We furthermore compared sleep quality scores before and after vaccination among four subgroups in Table 6. As per speculation, all participants with no ARs after the first vaccination including two subgroups indicated with red and blue lines in Figure 2 had significantly affected the sleep quality index (subgroup with no ARs after both vaccinations; subgroup with no ARs after first vaccination but ARs after the second one) (both *p* < 0.001). However, the last two subgroups failed to affect the sleep quality index (subgroup with ARs after the first vaccination but none after the second vaccination; subgroup with ARs after both vaccinations) (*p* = 0.139 and 0.299, respectively).

**TABLE 5:** Demographic characteristics among four subgroups to receive the coronavirus disease 2019 (COVID-19) vaccine.

	No ARs after both vaccinations (*n* = 233)	ARs after first vaccination but none after second one (*n* = 29)	No ARs after first vaccination but ARs after second one (*n* = 41)	ARs after both vaccinations (*n* = 42)	*P*-value
Demographics					
Age, years	36.61 ± 0.78	35.10 ± 2.39	39.19 ± 1.87	40.73 ± 2.02	0.113
No. of males, *n* (%)	163 (70)	25 (86.2)	36 (87.8)	33 (78.6)	0.042
No. of doctors, *n* (%)					
BMI, kg/m^2^	22.37 ± 0.22	22.19 ± 0.37	21.93 ± 0.30	22.81 ± 0.73	0.695
Marital status, *n* (% married)	161 (69.1)	16 (55.2)	34 (82.9)	30 (71.4)	0.093
Educational level (%)					
Below university	82 (35.2)	8 (27.6)	12 (29.3)	13 (31)	0.274
University	121 (51.9)	17 (58.6)	18 (43.9)	25 (59.5)	
Postgraduate	30 (12.9)	4 (13.8)	11 (26.8)	4 (9.5)	
Nightshift days per month	3.52 ± 0.31	4.10 ± 0.75	2.44 ± 0.41	4.69 ± 0.73	0.143
Professional title level, *n* (%)					
Junior	161 (69.1)	17 (58.6)	25 (61)	27 (64.3)	0.58
Mediate	42 (18)	6 (20.7)	7 (17.1)	6 (14.3)	
Senior	30 (12.9)	6 (20.7)	9 (22)	9 (21.4)	
Years of work, years	12.60 ± 0.77	13.69 ± 2.39	15.83 ± 1.89	15.81 ± 2.04	0.225
Current smoker, *n* (%)	5 (2.10)	1 (3.44)	2 (4.87)	2 (4.87)	0.432
Alcohol drinker, *n* (%)	6 (2.53)	1 (3.44)	2 (4.87)	2 (4.87)	0.411

Continuous data are presented as the means ± standard deviation (SD) and categorical data are presented as numbers with percentages in parentheses. Differences among four groups were examined using the Kruskal–Wallis *H*-test, one-way analysis of variance, Fisher’s exact test, or the χ2 test according to the data distribution.

**TABLE 6:** Comparison of sleep quality scores of healthcare workers measured using the Pittsburgh Sleep Quality Index (PSQI) assessment before and after vaccination among four subgroups to receive the coronavirus disease 2019 (COVID-19) vaccine.

	Baseline sleep quality	1 month following up	The change in sleep quality	*P*-value
No ARs after both vaccinations (*n* = 233)	4.45 ± 0.15	6.29 ± 0.16	1.83 ± 0.25	<0.001
ARs after first vaccination but none after second one (*n* = 29)	6.72 ± 0.28	5.55 ± 0.64	−1.17 ± 0.77	0.139
No ARs after first vaccination but ARs after second one (*n* = 41)	3.26 ± 0.38	8.43 ± 0.57	5.17 ± 0.66	<0.001
ARs after both vaccinations (*n* = 42)	6.33 ± 0.28	5.73 ± 0.44	−0.59 ± 0.56	0.299

The data are presented as the means ± SD. Scores obtained before and after 1 month following up were compared using the paired Student’s *t*-test.

## Discussion

To the best of our knowledge, there are no publications addressing sleep quality before and after the vaccination in relation to ARs to COVID-19 vaccination, yet the scientific community recently expressed the necessity for this kind of information (Lange et al., 2011; Prather et al., 2012). This study shows that the vaccine recipients experienced a poor subjective quality of sleep after vaccination. The baseline or follow-up sleep quality change was associated with the occurrence of ARs after vaccination.

In the present study, it was determined that serious AR frequency was relatively low in inactive vaccines. At least one AR was stated in 33.2% of the participants, which was consistent with that reported in the previous study (Zhang et al., 2021). However, the previous studies rarely provided information about sleep conditions pre- and post-COVID-19 vaccination and added sleep dysfunction as one AR after vaccination (Xia et al., 2020, 2021; Li et al., 2021; Zhang et al., 2021). Some other previous studies had reported that there were various other kinds of vaccine recipients who experienced some narcolepsy (Sarkanen T. et al., 2018; Sarkanen T. O. et al., 2018). Our result showed that HCWs experienced some reduced sleep quality after COVID-19 vaccination (Table 2).

In addition, we found that sleep quality before vaccination was associated with the occurrence of ARs after vaccination (Table 4). Although such interaction association between sleep quality and ARs following the administration of COVID-19 vaccines has not been investigated so far, its underlying mechanism remains unclear. One reason maybe was that psychological distress for concerns about the AR after vaccination might be either a cause of poor sleep dysfunction (Jaradat et al., 2020). Presumably, it was a circular dysfunctional process, where a higher level of distress was related to worse quality of sleep, which in turn led to higher levels of psychological stress (Strygin, 2011). Our previous study found that the subjective psychological stress scale was positively related to the total PSQI score (Zhao et al., 2020), which informed us to focus more on the prevention of psychological stress and sleep dysfunction.

It could be plausibly explained by sleep complaints and restricted sleep that have been identified as risk factors for various disorders (Meerlo et al., 2008) including cardiovascular diseases (Gangwisch et al., 2006) and psychiatric disorders (Ford and Kamerow, 1989). The decreased sleep quality after vaccination thus could increase the risk of these disorders as ARs. Many ARs after vaccination were belonging to the immunological responses (Nakayama, 2019). Thus, the last but important reason was the contribution of sleeping dysfunction to the dynamic variations in the immune system (Besedovsky et al., 2019). Previous research had linked sleep deprivation to the activation of sympathetic nervous activity and the hypothalamic-pituitary-adrenal axis, which suppresses antiviral responses and stimulates pro-inflammatory responses (Spiegel et al., 2002; Meerlo et al., 2008; Lange et al., 2011). Further research is needed to disentangle the cause-effect between sleep quality and ARs after vaccination.

The baseline level of sleep quality index from these two subgroups was lower than from the other two subgroups (4.45 ± 0.15 and 3.26 ± 0.38 vs. 6.72 ± 0.28 and 6.33 ± 0.28). Thus, the lower baseline level of sleep quality index had a small chance to suffer the ARs after vaccination in keeping with the findings in Table 4. The reason why the two subgroups with lower sleep quality index, meaning the more normal sleep quality, were more likely to experience more sleep quality change after vaccination was unclear. It may be related to the adaptations evident in many aspects of system homeostasis. For example, the circadian rhythm adaptation in the night shift worker affects their psychomotor performance, sleep quality, and subjective alertness (Boudreau et al., 2013). Additionally, sleep adaptation, the so-called “first-night effect,” is known to occur in healthy individuals (Song et al., 2013). Thus for people with normal baseline sleep quality, their circadian rhythm may be more easily disturbed by some new or short-term stress, while people with some sleep problems could avoid sleep disturbance from this stress. In Figure 2 as shown in red line subgroups, the people who experienced the more poor sleep quality between the two doses of injections would be more likely to have ARs after the second dose of injection, which suggested that the sleep quality changed after the first dose of injection may increase the risk to have ARs after the second dose of injection. In the two-dose vaccination programs, sleep quality monitoring may be helpful to predict and alleviate adverse events, even for people with normal baseline sleep quality or without adverse events after the first dose of vaccination.

Some limitations in the observational study should be discussed. First, although we adjusted for several common confounders, other factors such as exercise and dietary habits were not considered. Second, a more long-term follow-up study was needed to detect the change over time in sleep quality and whether the participants benefited more from the sleep interventions. Third, the sample in four subgroups was relatively less and may influence the statistical power. To further explore the potential role of change in sleep quality before and after vaccination on the difference in the chance of ARs after vaccination, a large sample randomized prospective interventional study would be needed.

## Conclusion

We should pay more attention to and effective monitoring of sleep quality for alleviating adverse events during the immunization program among the vaccine recipients during the epidemic situation.

## Data availability statement

The raw data supporting the conclusions of this article will be made available by the authors, without undue reservation.

## Ethics statement

The studies involving human participants were reviewed and approved by the Internal Review Board of the Institutional Ethics Committee of Sichuan Provincial People’s Hospital and were conducted in accordance with the Declaration of Helsinki. The patients/participants provided their written informed consent to participate in this study.

## Author contributions

XZ, GH, and JF had full access to all data in the study and took responsibility for the integrity of the data and accuracy of the data. XZ and XX designed the study. XY, YF, BilL, ZM, YL, and NX collected the data. XZ performed the statistical analysis. BL and XZ drafted the manuscript. All authors contributed to the article and approved the submitted version.
